# Statistical modeling of mRNP transport in dendrites: A comparative analysis of β‐actin and Arc mRNP dynamics

**DOI:** 10.1111/tra.12913

**Published:** 2023-08-06

**Authors:** Hyerim Ahn, Xavier Durang, Jae Youn Shim, Gaeun Park, Jae‐Hyung Jeon, Hye Yoon Park

**Affiliations:** ^1^ Department of Electrical and Computer Engineering University of Minnesota Minneapolis Minneapolis USA; ^2^ Department of Physics Pohang University of Science and Technology Pohang Republic of Korea; ^3^ Department of Physics and Astronomy Seoul National University Seoul Republic of Korea; ^4^ Asia Pacific Center for Theoretical Physics Pohang Republic of Korea; ^5^ Institute of Applied Physics Seoul National University Seoul Republic of Korea

**Keywords:** Arc, dendritic transport, Lévy walk, mRNA localization, β‐actin

## Abstract

Localization of messenger RNA (mRNA) in dendrites is crucial for regulating gene expression during long‐term memory formation. mRNA binds to RNA‐binding proteins (RBPs) to form messenger ribonucleoprotein (mRNP) complexes that are transported by motor proteins along microtubules to their target synapses. However, the dynamics by which mRNPs find their target locations in the dendrite have not been well understood. Here, we investigated the motion of endogenous β‐actin and Arc mRNPs in dissociated mouse hippocampal neurons using the MS2 and PP7 stem‐loop systems, respectively. By evaluating the statistical properties of mRNP movement, we found that the aging Lévy walk model effectively describes both β‐actin and Arc mRNP transport in proximal dendrites. A critical difference between β‐actin and Arc mRNPs was the aging time, the time lag between transport initiation and measurement initiation. The longer mean aging time of β‐actin mRNP (~100 s) compared with that of Arc mRNP (~30 s) reflects the longer half‐life of constitutively expressed β‐actin mRNP. Furthermore, our model also permitted us to estimate the ratio of newly generated and pre‐existing β‐actin mRNPs in the dendrites. This study offers a robust theoretical framework for mRNP transport, which provides insight into how mRNPs locate their targets in neurons.

## Graphical abstract

Localization of mRNA is essential for gene regulation in long‐term plasticity. The study investigated the motion of endogenous β‐actin and Arc mRNPs in mouse hippocampal neurons. The aging Lévy walk model effectively describes both mRNPs' transport in proximal dendrites, with a critical difference in aging time reflecting the longer half‐life of β‐actin mRNP. The study provides a theoretical framework for understanding how mRNPs locate their targets in neurons and estimate the ratio of newly generated and pre‐existing mRNPs in dendrites.

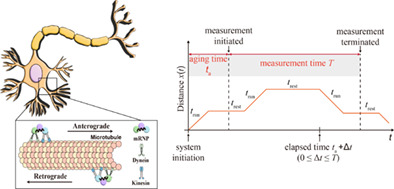

## 1 INTRODUCTION

Localization of mRNA and local protein translation are well known to regulate gene expression spatially and temporally.^1^, ^2^, ^3^ Inside cells, mRNA is transported in the form of mRNP complexes bound to several RBPs. In neurons, mRNP complexes are delivered to their target sites in a bidirectional movement by motor proteins along microtubules.^4^ Identifying the mechanism of mRNA localization is important for understanding gene expression underlying synaptic plasticity and long‐term memory formation.^5^, ^6^

The neuronal mRNP transport process can be described by a Lévy walk model, which consists of random walks with a finite velocity and rest periods in between.^7^ Recent studies have shown that Lévy walks are commonly observed in a variety of biological systems ranging from cells and animals to humans.^8^ However, Lévy walks of intracellular organelles or biomolecules are scarcely studied^9^; it is yet unknown how generally the aging Lévy walk model for neuronal mRNP transport^7^ can be applied to different mRNA species and neuronal activity states.

To investigate whether the Lévy walk model can be commonly applied to mRNA transport in neurons, we considered two types of genes: β‐actin and Arc, as representative mRNAs sorted in distinct mRNP granules. β‐actin (Actb) is an isoform of actin, one of the most abundant proteins in cells. In dendrites, actin cytoskeleton, composed of actin polymers and actin‐binding proteins, mediates the maintenance or change of dendritic spine morphology.^10^ The dendritic spines are created or eliminated by learning, and the long‐lasting changes in spine morphology are associated with synaptic plasticity and long‐term memory formation.^11^, ^12^ Arc (activity‐regulated cytoskeleton‐associated protein) is one of the immediate‐early genes (IEGs) that are transiently and rapidly activated in response to internal or external cellular signals.^13^, ^14^ Arc mRNA is transported to dendrites, localized to the stimulated synaptic regions, and translated into proteins.^15^ It is also well known that Arc has a role in the endocytosis of AMPA receptors and control of synaptic plasticity associated with long‐term potentiation, long‐term depression, and homeostatic plasticity.^16^

It has been suggested that some mRNA species are packaged together in mRNP granules, while others are sorted into separate granules.^17^, ^18^, ^19^ For instance, while Arc mRNA has been found to be co‐assembled with CaMKIIα and neurogranin mRNAs in the same mRNP granules,^17^ it was not colocalized with β‐actin mRNA.^20^ This implies that β‐actin and Arc mRNAs are sorted into different mRNP granules and transported independently. Nevertheless, it remains uncertain whether these heterogeneous mRNP granules utilize the same transport pathway or move via distinct mechanisms.

Advances in RNA imaging techniques have made it possible to visualize single endogenous β‐actin and Arc mRNP complexes in living neurons and tissues.^21^, ^22^, ^23^, ^24^ Using these techniques, we investigated the statistical properties of mRNP movements and found that an aging Lévy walk model can accurately describe the movement of both mRNPs. Through simulations of the aging Lévy walk model, we were able to estimate the aging time and the elapsed time from transport initiation to measurement initiation, and found that it matched the experimental results. This theoretical framework provides insight into the transport of mRNPs in dendrites, laying the groundwork for future studies on mRNA localization in neurons.

## 2 RESULTS

### 2.1 β‐actin and Arc mRNAs are sorted in distinct mRNP granules in neurons

**FIGURE 1:**
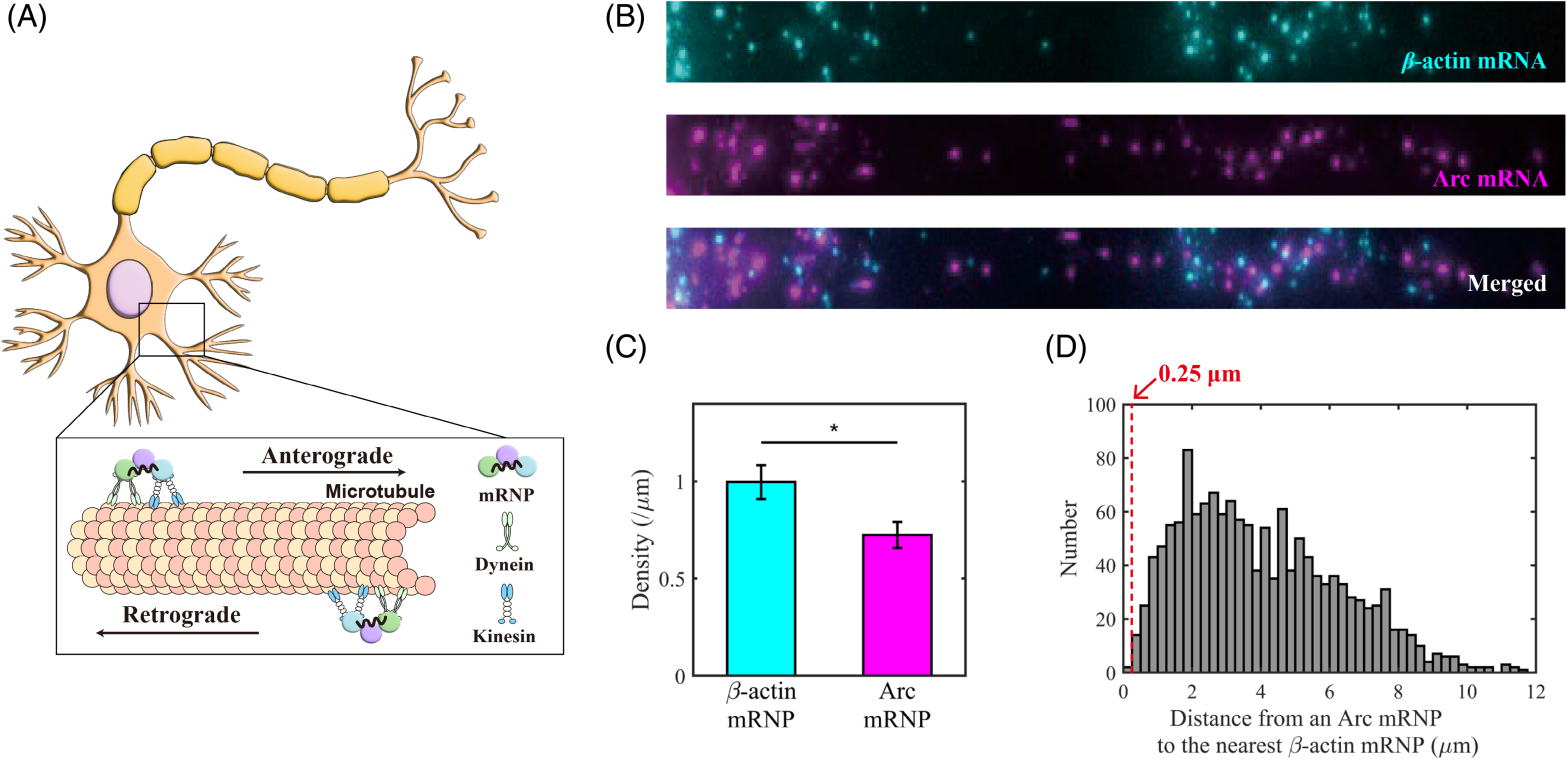
β‐actin and Arc mRNPs are packaged separately in dendrites. (A) Schematic of the motor‐driven transport of mRNP complexes in a neuron. An mRNP, in the form of a complex of mRNA bound to RBPs, is transported along microtubules by dynein and kinesin motor proteins. (B) Representative FISH images of β‐actin mRNA (cyan) and Arc mRNA (magenta) in the proximal dendrite. (C) Bar graph comparing the density of β‐actin and Arc mRNP per unit length (μm). In proximal dendrites, the density of β‐actin mRNP was significantly higher than that of Arc mRNP (* *p* < 0.05, pairwise *t* test; *n* = 54 dendrites). The error bars represent the standard error of mean (SEM). (D) Histogram of distances from an Arc mRNP to the nearest β‐actin mRNP (*n* = 1443 Arc mRNPs). The percentage of Arc mRNPs colocalized with β‐actin mRNP (within the threshold of 0.25 μm) was 0.14%.

In dendrites, mRNP particles travel along microtubules to locate their target sites in bidirectional motion driven by motor proteins such as dynein and kinesin (Figure 1A). To investigate whether β‐actin and Arc mRNAs are co‐assembled in the same mRNP granules, we performed single‐molecule fluorescence in situ hybridization (smFISH) (Figure 1B). We imaged β‐actin and Arc mRNAs in the proximal dendrites (< 50 μm from soma) of hippocampal neurons cultured from wild‐type mice at 11–12 days in vitro (DIV). Because Arc mRNA is expressed only in the presence of neuronal activity, we stimulated neurons with bicuculline, an antagonist of gamma‐aminobutyric acid (GABA) receptors. In each dendrite, we measured the number and position of β‐actin and Arc mRNPs at 40–60 min after the onset of the stimulation. We imaged 54 proximal dendrites, in which a total of 1871 β‐actin mRNPs and 1443 Arc mRNPs were observed. β‐actin mRNP had a significantly higher density of about 0.99 ± 0.09 particles/μm compared with Arc mRNP (0.72 ± 0.07 particles/μm) (Figure 1C). Then we measured the distance from each Arc mRNP to the nearest β‐actin mRNP (Figure 1D). Two particles were considered to be colocalized if their distance was shorter than 0.25 μm considering the optical diffraction limit. This threshold is also consistent with the criteria for colocalization in previous studies.^20^, ^25^ We found that only 2 out of 1443 Arc mRNPs were within 0.25 μm from the nearest β‐actin mRNP, and thus only 0.14% of all Arc mRNPs were colocalized with β‐actin mRNP. This is also consistent with a previous report showing β‐actin and Arc mRNAs do not colocalize with each other.^20^ This result suggests that β‐actin and Arc mRNAs reside in distinct mRNP populations possibly with different RBP and motor protein compositions.^26^

### 2.2 Transport of endogenous β‐actin and Arc mRNPs labeled with MS2/PP7 system

**FIGURE 2:**
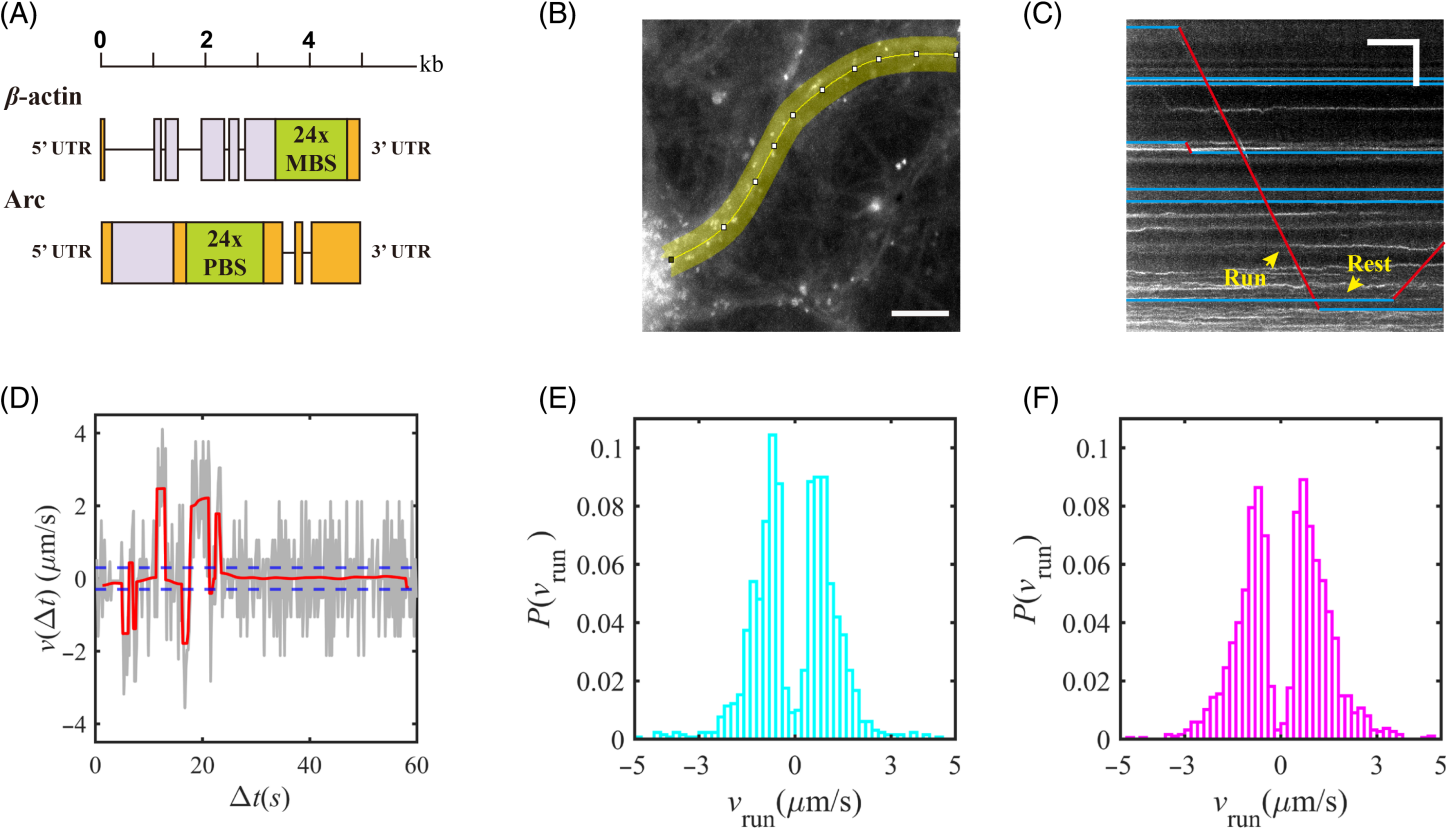
Transport of β‐actin and Arc mRNPs in live hippocampal neurons. (A) Schematic diagram of Actb‐MBS and Arc‐PBS knock‐in constructs (orange: untranslated region (UTR); purple: coding region; green: 24× MBS or PBS cassette). (B) Representative image of GFP‐labeled β‐actin mRNP in a live hippocampal neuron. The proximal dendritic region (yellow area) was straightened to generate a kymograph. Scale bar, 10 μm. (C) Kymograph generated from a straightened image of the dendrite in (B). The horizontal axis indicates the elapsed time and the vertical axis indicates the distance traveled. Trajectories of mRNPs are segmented into run (red lines) and rest (blue lines) phases. Scale bars, 10 s (horizontal axis), and 10 μm (vertical axis). (D) Local velocity of an mRNP particle over time (gray line) and filtered velocity (red line). Blue dashed lines indicate the threshold (±0.3 μm/s) to distinguish run and rest phases. (E, F) Histograms of run velocity distributions for β‐actin (E; cyan) and Arc (F; magenta) mRNPs.

Since β‐actin and Arc mRNAs are sorted in distinct mRNP granules, we next investigated whether they show different properties in their movement in dendrites. To monitor the motion of single mRNP particles in living hippocampal neurons, we used two genetically‐engineered mouse models expressing β‐actin and Arc mRNA labeled with green fluorescent proteins (GFPs). The Actb‐MBS KI mice were created by inserting 24 repeats of MS2 binding site (MBS) RNA stem‐loop into the 3′ untranslated region (UTR) of the β‐actin gene,^23^ while the Arc‐PBS KI mice utilized 24 repeats of PP7 binding site (PBS) RNA stem‐loop inserted into the 3′ UTR of the Arc gene^21^ (Figure 2A). To label the mRNA with GFPs, the Actb‐MBS KI mouse was crossed with a transgenic mouse expressing MS2 capsid protein (MCP) fused with GFP (MCP mouse) to obtain the MCP × MBS hybrid mouse,^24^ while the Arc‐PBS KI mouse was crossed with a mouse expressing PP7 capsid protein (PCP) fused with GFP (PCP mouse) to generate the PCP × PBS mouse.^22^ In the homozygous MCP × MBS and PCP × PBS mice, all endogenous β‐actin and Arc mRNAs, respectively, were labeled with up to 48 GFPs.

We cultured hippocampal neurons from the MCP × MBS and PCP × PBS mice, stimulated the neurons with bicuculline at 12–15 DIV, and imaged the movement of mRNPs for 1 min at 40–70 min after the onset of stimulation. After imaging, we selected dendritic regions of interest and made a straightened image for each dendrite (Figure 2B). Kymographs were generated from the straightened images and used to obtain one‐dimensional trajectories of individual mRNP particles over time (time interval: 0.2 s) (Figure 2C). For β‐actin mRNP, we imaged 13 cells in 11 batches of neuron cultures and analyzed 2910 β‐actin mRNP trajectories. For Arc mRNP, 54 cells in 20 batches were imaged and 3185 Arc mRNP trajectories were analyzed. To analyze the dynamics of mRNP particles, we calculated the local velocity at each time step. These local velocity profiles (gray curve in Figure 2D) were smoothed using iterative bilateral filtering which replaced the velocity value with a local weighted average ${\hat{v}}_{i}$ (red curve in Figure 2D). By considering a velocity threshold $v_{\text{threshold}}=0.3$ μm/s, the trajectories were segmented into the rest phase when $\left|{\hat{v}}_{i}\right|<v_{\text{threshold}}$ and the run phase otherwise (Figure 2D). The velocity of each run was evaluated according to $v_{\mathrm{run}}=\frac{\Delta x_{\mathrm{run}}}{\Delta t_{\mathrm{run}}}$, where $\Delta x_{\mathrm{run}}$ is the displacement and $\Delta t_{\mathrm{run}}$ is the duration of each run. The velocity distributions of β‐actin and Arc mRNPs are shown in Figure 2E,F.

Anterograde and retrograde average velocities were calculated as the mean of the positive and negative velocities of the run phases, $v_{\mathrm{run}}$. For β‐actin mRNP, the anterograde and retrograde average velocities were 1.0 μm/s and −1.1 μm/s, respectively (Figure 2E). Notably, there was no directional preference; both anterograde and retrograde runs occurred with the same probability. In contrast, Arc mRNP exhibited a bias toward anterograde runs (54%) rather than retrograde runs (46%). The anterograde average velocity of Arc mRNP was 1.3 μm/s and the retrograde average velocity was ‐ 1.1 μm/s (Figure 2F).

### 2.3 Random walk properties of β‐actin and Arc mRNPs

**FIGURE 3:**
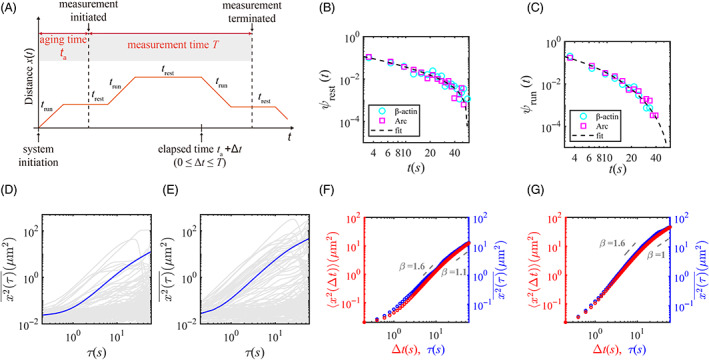
Characteristics of individual mRNP transport in proximal dendrites. (A) Schematic diagram showing the movement of an mRNP particle. Aging time $t_{a}$ is the time from the transport initiation to the measurement initiation. The experimental measurement time *T* is 60 s. $t_{\mathrm{run}}$ and $t_{\text{rest}}$ are the duration of the run and rest phases, respectively. (B, C) The rest time PDF $\psi_{\text{rest}}$ (B) and run time PDF $\psi_{\mathrm{run}}$ (C) for β‐actin (cyan circles) and Arc (magenta squares) mRNPs with fitting curves (black dashed lines). (D, E) TA MSD curves of experimental trajectories of 100 randomly selected β‐actin (D) and Arc (E) mRNPs (gray lines). The thick blue lines indicate the average of all TA MSD curves. (F, G) EA MSD (red) and TA MSD (blue) curves of β‐actin (F) and Arc (G) mRNPs.

Based on our observation of mRNP movement, we reasoned that a random walk model consisting of ballistic run and static rest phases^7^ can be applied to both β‐actin and Arc mRNPs. In this model, we refer to aging time as the elapsed time from the starting of mRNP movement in the dendrite to the initiation of measurement (Figure 3A). From the mRNP trajectories, we obtained the probability density functions (PDFs), that is, the distributions of duration times of the rest and run events, for β‐actin and Arc mRNPs (Figure 3B,C). An important finding is that the statistics of run and rest duration times were almost identical for β‐actin and Arc mRNPs. Although β‐actin and Arc mRNAs were sorted in different mRNP populations, their movements in proximal dendrites had similar characteristics with long rest times (Figure 3B) and relatively short run times (Figure 3C). This means that the mRNP transport in dendrites can be quantified, regardless of the mRNA species, by a single dynamic model with the same PDFs. Based on our previous study,^7^ we modeled the rest time PDF with a power‐law (normalized) distribution

(1) $$\psi_{\text{rest}}\left(t\right)=\frac{\alpha_{\text{rest}}}{{\left(t+1\right)}^{1+\alpha_{\text{rest}}}},$$

where $\alpha_{\text{rest}}>0$ is the power‐law exponent of the distribution, and the run time PDF with a truncated power‐law distribution

(2) $$\psi_{\mathrm{run}}\left(t\right)=\frac{1}{Ɲ}\frac{e^{-t/\tau_{r}}}{{\left(t+1\right)}^{1+\eta_{\mathrm{run}}}},$$

where $\eta_{\mathrm{run}}>0$ is the power‐law exponent, $\tau_{r}$ is the characteristic time for the run phase, and $Ɲ=e^{1/\tau_{r}}\Gamma \left(-\eta_{\mathrm{run}},1/\tau_{r}\right)/\tau_{r}^{\eta_{\mathrm{run}}}$ is the normalization factor. The two power‐law exponents $\alpha_{\text{rest}}$ and $\eta_{\mathrm{run}}$ and the characteristic time $\tau_{r}$ were extracted by fitting the experimental data of rest and run time PDFs with Equation (1) and (2), respectively. Here, we advanced this fitting procedure by exploiting our new theory that enables one to correctly infer the fit parameters under the limits of the finite observation time window and the time resolution.^27^ The dashed line in Figure 3B depicts the resultant best fit to the rest time PDF with $\alpha_{\text{rest}}$ = 0.24. Similarly, the dashed line in Figure 3C shows the fit using a truncated power‐law with $\eta_{\mathrm{run}}$ = 0.18 and $\tau_{r}$ = 12.6 s. Using these parameters, we constructed and simulated the aging Lévy walk model for the movement of single mRNP particles following similar approaches in our previous study.^7^

By analyzing each mRNP trajectory, we investigated the time‐averaged mean squared displacement (TA MSD) curves $\bar{x^{2}\left(\tau \right)}$ where the τ is the lag time and *T* is the total measurement time for each mRNP.^28^, ^29^

(3) $$\bar{x^{2}\left(\tau \right)}=\frac{1}{T-\tau }\int_{0}^{T-\tau }d\tau_{0}{\left[x\left(\tau +\tau_{0}\right)-x\left(\tau_{0}\right)\right]}^{2}.$$

TA MSD is defined as the MSD obtained from the moving average of displacements of a single trajectory for given lag time τ. The average curves of $\bar{x^{2}\left(\tau \right)\;}$ for all trajectories indicated by the thick blue lines have a similar shape but different scales for β‐actin and Arc mRNPs (Figure 3D,E, and Figure S1). Additionally, the ensemble‐averaged mean squared displacement (EA MSD) curves $\left\langle x^{2}\left(∆t\right)\right\rangle$ (red) were also plotted compared with the averaged TA MSD curves (blue) in Figure 3F,G.

(4) $$\left\langle x^{2}\left(∆t\right)\right\rangle =\frac{1}{N}\sum_{i=1}^{N}{\left[x_{i}\left(\Delta t\right)-x_{i}\left(0\right)\right]}^{2}.$$

The trajectory $x_{i}\left(\Delta t\right)$ of each mRNP particle is denoted by the index i, and N means the total number of observed mRNPs. EA MSD is calculated as the MSD for the displacement from the initial position $x_{i}\left(0\right)$ to the $x_{i}\left(∆t\right)$ of all particles. For both β‐actin and Arc mRNPs, the two MSD curves (blue & red) tend to be almost identical during the measurement time *T* = 60 s (Figure 3F,G). According to the TA MSDs, the MSD exponent β of $\bar{x^{2}\left(\tau \right)}$ (~$\tau^{\beta }$) determines the type of anomalous diffusion: the mRNP transport is super‐diffusive if β > 1, Brownian if β = 1, and sub‐diffusive if 0<β<1. ^29^, ^30^ In both MSD curves, it was shown that the mRNP transport was super‐diffusive with the anomalous exponent β≈1.6 (>1) for τ<10 s, and after 10 s, it became normal diffusion with the exponent β≈1. ^31^ The observed transport behaviors are also consistent with the previous study on β‐actin mRNP transport in dendrites.^7^

### 2.4 An aging Lévy walk model with estimated aging time

By analyzing experimental data for β‐actin and Arc mRNP, we determined all the parameters to apply the Lévy walk model to the transport kinetics of each mRNP. Using these parameter values, we performed simulations of our aging Lévy walk model (Figure S2), generating 50 000 trajectories for each case of β‐actin and Arc mRNP. As the simulated trajectories lacked the fluidic noise present in actual experimental trajectories,^32^ we incorporated this effect by randomly extracting fluidic noise from the experimental trajectory and superimposing it onto the simulated trajectories.

**FIGURE 4:**
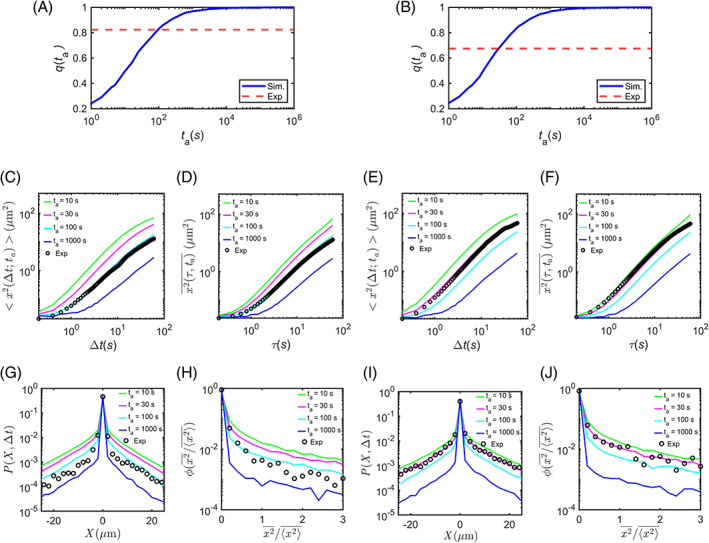
Estimation of aging time through comparison of experimental data and simulation of aging Lévy walk with rest model. (A, B) Fraction of the simulated trajectories showing no run during [$t_{a}$, $t_{a}$+*T*] as a function of $t_{a}$ for β‐actin (A) and Arc (B) mRNPs. Blue solid lines represent the simulation curves and red dashed lines indicate the corresponding experimental values. (C, E) Comparison of the EA MSD (black circles) of the experimental data for β‐actin (C) and Arc (E) mRNPs to the EA MSD curves of the simulation data with various aging times $t_{a}$= 10, 30, 100, and 1000 s (green, magenta, cyan, and blue curves, respectively). (D, F) Comparison of the TA MSD (black circles) of the experimental data for β‐actin (D) and Arc (F) mRNPs to the TA MSD curves of the simulation data. (G, I) Comparison of the aged probability density function $P\left(X,∆t\right)$ of the experimental data for β‐actin (G) and Arc (I) mRNPs to $P\left(X,∆t\right)$ of the simulation data. (H, J) Comparison of the scatter distribution function $\phi \left(\bar{x^{2}}/\left\langle \bar{x^{2}}\right\rangle \right)$ of the TAMSD of the experimental data for β‐actin (H) and Arc (J) mRNPs to those of the simulation data.

In the trajectories of experimental data, we noted that the rest phases were overwhelming the run phases. The percentage of trajectories without any run event during the measurement time [$t_{a},$ $t_{a}$+*T*] was about 82% for β‐actin mRNP and 68% for Arc mRNP. We also plotted the fraction of the simulated trajectories that did not have any run phase in [$t_{a},$ $t_{a}$+*T*] as a function of aging time $t_{a}$ and inferred the aging time of our experimental data by finding the crossing point of the curves (Figure 4A,B). The mean aging time was estimated to be about 100 s for β‐actin mRNP and 30 s for Arc mRNP. The aging time of Arc mRNP was shorter than that of β‐actin mRNP, which could be attributed to a shorter half‐life of Arc mRNP (~60 min)^21^ compared with that of β‐actin mRNP (~9 h).^33^, ^34^

To crosscheck whether the aging Lévy walk model explains well the motions of β‐actin and Arc mRNP transport, we compared the experimental MSD curves to the simulated MSDs with varying aging times of 0, 30, 100, and 1000 s. For β‐actin, the MSD curves showed good agreement between the experimental data and the simulation when using an aging time of $t_{a}=100$ s for both EA MSD and TA MSD (Figure 4C,D). Similarly, for Arc, the simulation using the estimated aging time of $t_{a}$ = 30 s was able to accurately explain the experimental EA MSD and TA MSD (Figure 4E,F). However, when analyzing the aged PDF $P\left(X,\Delta t=30\;\text{s}\right)$ (Figure 4G) and the scatter distributions of $\bar{x^{2}}$/$\bar{\left\langle x^{2}\right\rangle }$ (Figure 4H), β‐actin mRNP data deviated from the simulation results with $t_{a}=100$ s. In contrast, the results for Arc mRNP data still showed strong agreement with the simulation with $t_{a}$ = 30 s (Figure 4I,J). These results suggest that our theoretical model effectively explains the general dynamics of mRNP transport in dendrites, but further investigation is needed to determine the aging time of β‐actin mRNP.

### 2.5 Estimation of the ratio between newly formed and pre‐existing β‐actin mRNP

When neurons are stimulated, both β‐actin and Arc mRNA are newly transcribed.^21^, ^24^ However, unlike Arc mRNA which is only expressed transiently in response to external stimuli, β‐actin mRNA is constitutively expressed even in the absence of neuronal activity. Consequently, β‐actin mRNPs in dendrites consist of two distinct populations: those that pre‐exist before stimulation and those that are newly generated in response to stimulation. This could be one of the reasons why the density of β‐actin mRNP in the dendrite is higher than that of Arc mRNP (Figure 1C).

**FIGURE 5:**
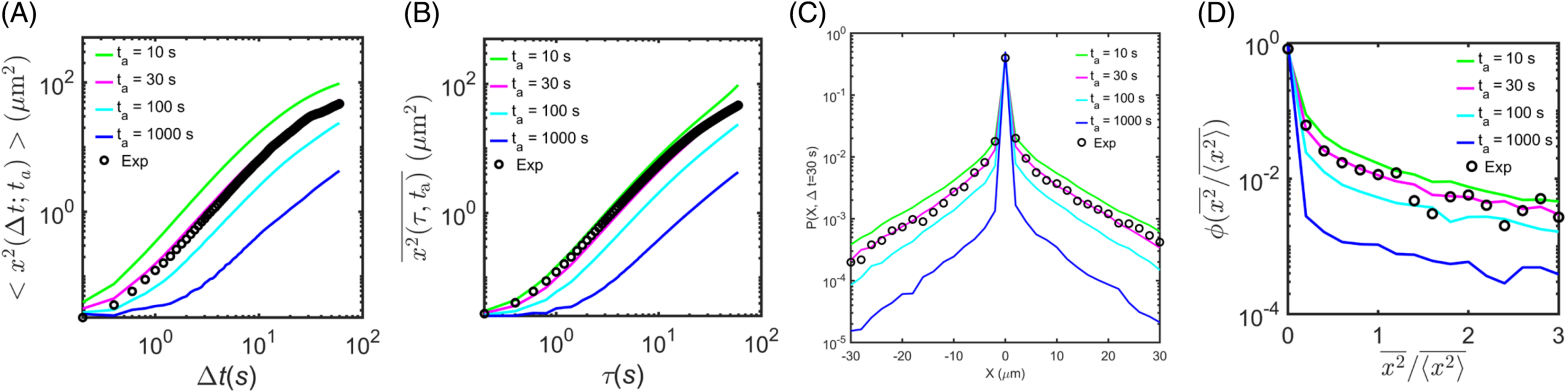
β‐actin mRNPs consisting of two populations, pre‐existing before stimulation and newly generated after stimulation. (A) EA MSD curves of the experimental data for β‐actin mRNP (black circles) and the simulation data consisting of two populations with different ratios between newly formed mRNP ($t_{a}$ = 30 s) and pre‐existing mRNP ($t_{a}$ = 11 min). (B) TA MSD curves of the experimental data for β‐actin mRNP (black circles) and the simulation data. (C) The aged probability density functions $P\left(X,∆t\right)$ of the experimental data for β‐actin mRNP (black circles) and the simulation data. (D) The scatter distribution function $\phi \left(\bar{x^{2}}/\left\langle \bar{x^{2}}\right\rangle \right)$ of the TA MSD of the experimental data for β‐actin mRNP (black circles) and the simulation data. The ratios between newly formed and pre‐existing mRNPs are 1:9, 3:7, 5:5, and 7:3 (green, magenta, cyan, and blue curves, respectively). The simulation curve with a ratio of 3:7 (magenta) shows the best agreement with the experimental data.

We observed that the aged PDF $P\left(X,\Delta t\right)$ and the scatter distribution of $\bar{x^{2}}$/$\bar{\left\langle x^{2}\right\rangle }$ of β‐actin mRNP did not align with simulation results using a single mean aging time (Figure 4G,H), unlike the Arc mRNP case (Figure 4I,J). Given that the aging time is associated with mRNA production, we considered that the β‐actin mRNP population consisted of two groups with different aging times. To estimate the average aging time of pre‐existing β‐actin mRNP, we analyzed mRNP movement in unstimulated neurons probed at the time interval of 0.2 s. We found that the TA MSD curve of β‐actin mRNP in unstimulated neurons (blue) was lower than that in stimulated neurons (cyan) (Figure S3A). The percentage of stationary β‐actin mRNP in unstimulated neurons was 88% (red dashed line, Figure S3B), which was higher than 82% in stimulated neurons (Figure 4A). By comparing with the simulation data (blue solid line), we estimated that the average aging time of pre‐existing β‐actin mRNP was approximately 11 min (Figure S3B). In stimulated neurons, we assumed that newly generated β‐actin mRNP had the same average aging time of 30 s as Arc mRNP. Then, we ran simulations with different ratios between the newly generated and pre‐existing mRNP populations, ranging from 1:9, 3:7, 5:5, to 7:3. A ratio of 3:7 gave the best agreement with the β‐actin experimental data (Figure 5). In particular, both the PDF $P\left(X,\Delta t\right)$ (Figure 5C) and scatter distribution of $\bar{x^{2}}$/$\bar{\left\langle x^{2}\right\rangle }$ (Figure 5D) were significantly improved compared with the results obtained when only one population was considered (Figure 4G,H).

We also conducted simulations with various aging times of pre‐existing mRNP from 10 min (Figure S4A), 1 h (Figure S4B), to 10 h (Figure S4C). As the aging time increased, the proportion of immobile mRNP increased, resulting in more trajectories without the run phase dominating the simulation results. When the aging time of pre‐existing mRNP was set to be longer than 10 min, the simulation results became less sensitive to the aging time. The best agreement between experimental data and simulation was achieved with a 3:7 ratio of newly generated to pre‐existing β‐actin mRNP for all three cases of 10 min, 1 h, and 10 h (Figure S4). In summary, by utilizing the aging Lévy walk model, we were able to estimate the ratio of newly formed and pre‐existing mRNPs, which could not be directly measured experimentally.

## 3 DISCUSSION

In this study, we characterized and compared the statistical properties of dendritic transport of β‐actin and Arc mRNPs. We performed two‐color FISH for β‐actin and Arc mRNA to show that they do not colocalize in proximal dendrites (Figure 1). This means that the two mRNA species with different biological roles are sorted in distinct mRNP populations and transported separately. We then used genetically‐modified mice to visualize single endogenous β‐actin^24^ and Arc mRNPs^22^ in real time in live neurons. The motion of mRNP particles in proximal dendrites was detected, filtered, and classified by a velocity threshold criterion, showing that their trajectories were clearly divided into run and rest phases (Figure 2).

The transport dynamics of these two types of mRNPs are well described by a simple aging Lévy walk model with common parameters. The parameters applied to our Lévy walk model were determined by the distributions for rest time and run time obtained from the experimental data of β‐actin and Arc mRNPs (Figure 3B,C). By applying these determined parameters, we simulated the Lévy walk model for the two types of mRNPs, reflecting the statistical properties of each mRNP. The main difference between β‐actin and Arc mRNPs turned out to be the aging time, which was affected by the half‐life of the mRNA. The aging time for each mRNP was estimated from the fraction of the stationary trajectories by comparing the simulation result of the aging Lévy walk model with the experimental data.

By examining key dynamic features such as EA MSD, TA MSD, $P\left(X,\Delta t\right)$, and $\phi (\bar{x^{2}}$/$\bar{\left\langle x^{2}\right\rangle }),$ ^28^ we found that the Lévy walk model with the estimated aging time showed an excellent agreement with the experimental data of Arc mRNP but not so well with that of β‐actin mRNP (Figure 4). Unlike Arc mRNA, which is generated only after neuronal stimulation, β‐actin mRNA is always present in dendrites. By considering both old and new populations of β‐actin mRNPs, we were able to match the simulation data with the experimental results. Using the aging Lévy walk model, we predicted a 3:7 ratio of newly synthesized to pre‐existing β‐actin mRNPs in proximal dendrites (Figure 5).

We expect our aging Lévy walk model to provide a quantitative understanding of the transport mechanism by which mRNPs find their target locations in neurons. This model accurately predicted that there are more heterogeneous populations of β‐actin mRNP than Arc mRNP through the concept of the aging time. The aging time is not the biological age of the mRNP. It is a physical parameter denoting the time from the first run of the mRNP to the detection in the imaging experiment. Although the aging time is partially dependent on the age of the mRNP, it is much shorter than the half‐life of the mRNP in the proximal dendrite. In distal dendrites, most of mRNPs were stationary, reflecting a much longer aging time (data not shown). Our proposed aging Lévy walk model is meaningful in that it is the simplest theoretical model that can explain the transport mechanism of different mRNP species. Furthermore, this model allows us to predict the ratio of newly generated and pre‐existing mRNP populations, which is difficult to obtain experimentally. This work establishes a robust biophysical model for mRNP transport, which sheds light on the general target search mechanism of mRNPs in neurons.

## 4 MATERIALS AND METHODS

### 4.1 Mouse hippocampal neuron culture

All animal experimental procedures were performed according to the methods approved by the Institutional Animal Care and Use Committee (IACUC) at Seoul National University. Mouse hippocampal neurons were dissected from the brain of 0.5–2.5‐day‐old mouse pups expressing either GFP‐labeled Arc mRNA or β‐actin mRNA following the procedures described previously.^35^ The tube containing hippocampi was moved into a laminar flow hood and cold neural dissection solution (NDS) was removed until 2 mL of NDS remained. Then, 200 μL of 10× trypsin (Gibco) was added, and hippocampi were incubated at 37°C for 15 min. Then, trypsin was removed and 3 mL plating medium (PM) was added to triturate until most of the large chunks were dissociated. About 200 μL of dissociated hippocampal neurons were seeded on the poly‐D‐lysine (PDL, sigma‐Aldrich P7886) coated glass‐bottom dishes (SPL 100350). The seeded dishes were incubated at 37°C with 5% CO_2_ for 4 h. Neurobasal‐A medium (Gibco) supplemented with B‐27 (Gibco, 17 504 044), 1× GlutaMax (Gibco, 35 050 079), and Primocin (InvivoGen, ant‐pm‐1) was added to grow neurons at 37°C with 5% CO_2_. Neurons were incubated for 12–15 days prior to imaging.

### 4.2 Fluorescence in situ hybridization (FISH) and colocalization analysis

RNAscope® (ACD) in situ hybridization assay was performed according to the manuals provided by ACD. Neurons of 0.5–2.5‐day‐old C57BL/6 wild‐type pups were cultured on 18‐mm coverslips. Neurons at 11–12 DIV were stimulated by 50 μM bicuculline, blocking GABAergic receptors. At 40–60 min after stimulation, neurons were fixed with 4% paraformaldehyde. Permeabilization was performed using 100%, 70%, and 50% ethanol in series. Tween 20 and protease were used to block non‐specific binding sites and protein degradation. Arc and β‐actin mRNAs were hybridized with RNAscope® probes labeled with Atto 550 and Atto 647 fluorophores (ACD), respectively. After hybridization, coverslips with neurons were mounted on slide glasses with ProLong diamond antifade mountant (Invitrogen, P36961) for microscope imaging and long‐term storage. A fluorescence microscope system (Olympus, IX83) was utilized with a 150×/1.45 NA oil objective lens (Olympus, UAPON 150XOTIRF), an electron multiplying charge‐coupled device (EMCCD) camera (Andor, iXon Ultra 897), a white light emitting diode (LED) light source (Lumencor, SOLA), and Cy3 (Chroma, 49 004) and Cy5 (Chroma, 49 006) filter cubes.

To analyze FISH images, we compared the images from all channels, determined the dendritic regions for analysis, and straightened them using Fiji software.^36^ To determine the three‐dimensional positions of β‐actin and Arc mRNPs, TrackNTrace software^37^ was used. Particles in the z‐stack (separated by 0.25 μm) images were detected by the normalized cross‐correlation method with the parameters of PSF‐sigma 0.75, CorreThreshold 0.50, distanceFactor 3 for β‐actin mRNP, and PSF‐sigma 0.75, CorreThreshold 0.45, and distanceFactor 3 for Arc mRNP. The results were refined by the GPU‐Gauss MLE method with 10 iterations. If the same particle was detected in at least three consecutive z‐stack images at nearly identical positions within three pixels, it was regarded as a single mRNP particle. The three‐dimensional position of each mRNP was determined by the position of the brightest spot among those spots detected in the consecutive z‐stack images.

For colocalization analysis of β‐actin and Arc mRNP, the three‐dimensional distance from each Arc mRNP to the nearest β‐actin mRNP was calculated. If the distance between the nearest neighbor β‐actin and Arc mRNP was within 0.25 μm, the two mRNPs were considered to be colocalized. To measure the density of β‐actin and Arc mRNPs in dendrites, we counted the number of mRNPs, divided it by the length of the dendrite, and averaged the mRNP density in each dendrite.

### 4.3 Live‐cell imaging of endogenous β‐actin and Arc mRNP

For live‐cell imaging, hippocampal neurons cultured from the MCP × MBS^24^ and the PCP × PBS mice^22^ were used to image β‐actin and Arc mRNA, respectively. Neurons at 12–15 DIV were stimulated with 50 μM bicuculline (Tocris, 0130) for 20 min. Then, the neurons were washed with warmed HEPES‐buffered saline (HBS) medium (119 mM NaCl (Merck, 106404.1000), 30 mM D‐glucose (Sigma‐Aldrich, G7021), 20 mM HEPES (Gibco, 15 630–080), 5 mM KCl (Invitrogen, POTC01), 2 mM CaCl_2_ (Invitrogen, CBC002), 2 mM MgCl_2_ (Invitrogen, CBM001) at pH 7.4). The neurons were transferred to a wide‐field fluorescence microscope and incubated at 37°C for 20 min while multiple fields of view containing dendrites of interest were selected. Time‐lapse images were taken at 40–70 min after the onset of bicuculline stimulation at 5 frame per second (fps) for 1 min. An inverted microscope (Olympus IX‐73) was used with a 150×/1.45 NA oil immersion objective (Olympus, UAPON150XOTIRF), an EMCCD camera (Andor, iXon Ultra DU‐897 U), an LED light source (Lumencor, SOLA), and a motorized stage (ASI, MS‐2000‐500 XYZ).

### 4.4 Image analysis of *β*‐actin and Arc mRNP in live neurons

To analyze β‐actin and Arc mRNP movement, the dendritic regions of interest were selected and straightened using Fiji software.^36^ Using custom MATLAB code, we generated a kymograph from the straightened time‐lapse images and performed image analysis. Based on the manually selected trajectories in the kymograph, local maxima were detected within 5 pixels, and the one‐dimensional centroid (mean) was calculated for sub‐pixel localization of each particle. Each mRNP trajectory was divided into run and rest phases according to the local velocity. We calculated the local velocity $v_{i}=\frac{x_{i+1}-x_{i}}{\mathrm{\Delta t}}$ (Figure 2D) and smoothed the resulting velocity profile using an iterative bilateral filter.^38^ At the *n*th application of the filter, the velocities were averaged according to their proximity in time and in velocity value such that ${\hat{v}}_{i,n}=\frac{1}{\Theta }\;\sum_{j=-6}^{6}{\hat{v}}_{i,n-1}G_{\sigma_{s}}\left(j\right)G_{\sigma_{v}}\left(\left|v_{i-j}-v_{i}\right|\right)$ where $G_{\sigma }\left(x\right)$ is a Gaussian weight of variance σ, Θ is the normalization factor $\Theta =\sum_{j=-6}^{6}G_{\sigma_{s}}\left(j\right)G_{\sigma_{v}}\left(\left|v_{i-j}-v_{i}\right|\right)$, and ${\hat{v}}_{i,n}$ is the velocity at time step *i* after n applications of the filter. The value of the variances are given by $\sigma_{s,n}=\sigma_{s,n-1}+\frac{0.15}{n^{2}}$ with the initial condition $\sigma_{s,1}=0.078$, and $\sigma_{v,n}=0.05+\frac{\sigma_{v,n-1}-0.05}{n^{\frac{1}{10}}}$ with the initial condition $\sigma_{v,1}=8.$ With these variances, for the first few iterations, the filter behaved as a Gaussian filter, mostly averaging velocity close in time. After a larger number of iterations, it considered velocity close in value. The filtering process stopped when $\left|{\hat{v}}_{i,n}-{\hat{v}}_{i,n-1}\right|<0.01$ for all time steps i after the *n*th iteration. The resulting velocity profile ${\hat{v}}_{i}$ allowed automatic segmentation of rest and run phases using the velocity threshold $v_{\text{threshold}}$; when $\left|{\hat{v}}_{i}\right|<v_{\text{threshold}}$, it was regarded to be in the rest phase, otherwise it was regarded to be in the run phase. The velocity of each run phase (Figure 2E,F) was calculated by dividing the total displacement during a run by its duration. After identifying the status of phases from all experimental trajectories, we obtained the experimental PDFs of duration times of the run and rest phases.

For our computational study, we modeled the two experimental PDFs using power‐law‐based fit functions. We employed our recently developed theoretical method for fitting the PDFs to extract the parameter values in the presence of the following two experimental limits^27^: (a) the finite observation time window which was the duration of the experiment and was responsible for the hard cut‐off around 60 s, and (b) the temporal resolution which was the minimal number of time steps needed to decide in which state the mRNP was. It turns out that the experimentally determined PDFs $\psi_{\mathrm{ext}}\left(t\right)$ differ from the original PDFs $\psi \left(t\right)$ by the experimental setup ${\hat{L}}_{\mathrm{ext}}$ via the relation of $\psi_{\mathrm{ext}}\left(t\right)={\hat{L}}_{\mathrm{ext}}\psi \left(t\right)$. The two required parameters in ${\hat{L}}_{\mathrm{ext}}$ were the time window parameter T=57.4 and the resolution parameter which had been estimated to 7 data points.^27^ The best fitting values were obtained using the least square method onto the aggregated data of β‐actin and Arc mRNPs: the fitting distribution was ${\hat{L}}_{\exp}\left(\frac{\alpha_{\text{rest}}}{{\left(t+1\right)}^{1+\alpha_{\text{rest}}}}\right)$ for the rest time PDF, and ${\hat{L}}_{\exp}\left(\frac{{\tau_{r}}^{\eta_{\mathrm{run}}}}{e^{\frac{1}{\tau_{r}}}\Gamma \left(-\eta_{\mathrm{run}},\frac{1}{\tau_{r}}\right)}\frac{e^{-\frac{t}{\tau_{r}}}}{{\left(t+1\right)}^{1+\eta_{\mathrm{run}}}}\right)$ for the run time PDF, respectively.

### 4.5 Lévy walk model and numerical simulations

The Lévy walk with rests is a random walk model consisting of an alternation of run and rest phases.^7^ The run is the phase where a random walker moves with a constant velocity, while the rest is the state where the walker temporally stops. The two dynamic phases randomly switch each other, which is governed by respective sojourn time PDFs (i.e., distributions)^7^ (Figure 3A). In our model, based on our experimental data, we considered the rest time PDF to be a power‐law distribution Equation (1) and the run time PDF to be an exponentially truncated power‐law Equation (2). For numerical simulations, we used the parameters extracted from the rest and run time PDFs of our experimental data.

At time t=0, all the generated trajectories started from x=0 with a run whose constant velocity was either $v_{\text{antero}}$ with probability $p_{\text{antero}}$ or $v_{\text{retro}}$ with probability $1-p_{\text{antero}}$. At the beginning of a state, its duration was generated from the corresponding PDF using the inverse transform sampling method.^39^ The position was updated at each time step Δt=0.2 s until the end of the state. From the aging time $t=t_{a}$ to the end of the time window $t=t_{a}+T$, the position was recorded and we superposed the noise extracted from experimental data (randomly chosen trajectories being entirely in the rest phase). We simulated $N=10^{5}$ trajectories with a sampling time step Δt=0.2s and an observation time window T=60s.

## AUTHOR CONTRIBUTIONS

Jae‐Hyung Jeon and Hye Yoon Park conceived and designed the project. Hyerim Ahn, Jae Youn Shim, and Gaeun Park performed the experiments and data analysis. Xavier Durang developed theoretical models and performed simulations and data analysis. Hyerim Ahn, Xavier Durang, Jae‐Hyung Jeon and Hye Yoon Park wrote the paper.

## FUNDING INFORMATION

This work was supported by the National Research Foundation (NRF) of Korea, Grants No. RS‐2023‐00245195 (Xavier Durang); No. 2017K1A1A2013241, No. 2020R1A2C4002490, and No. 2021R1A6A1A10042944 (Jae‐Hyung Jeon); No. 2020R1A2C2007285 and the International Research Scholar Award from the Wellcome Trust (208468/Z/17/Z) (Hye Yoon Park).

## CONFLICT OF INTEREST STATEMENT

The authors declare no competing interests.

### PEER REVIEW

The peer review history for this article is available at https://www.webofscience.com/api/gateway/wos/peer-review/10.1111/tra.12913.

## Supporting information


**Data S1.** Supporting information.

## Data Availability

The data sets generated and analyzed in this study are available in the figshare repository, doi:10.6084/m9.figshare.23804964 and doi:10.6084/m9.figshare.23804997 for β‐actin mRNP and doi:10.6084/m9.figshare.23805003, doi:10.6084/m9.figshare.23805009, and doi:10.6084/m9.figshare.23805927 for Arc mRNP.
