# Supplementary material for: Statistical modeling of mRNP transport in dendrites: A comparative analysis of β‐actin and Arc mRNP dynamics
Source: Traffic. 2023 Aug 6;24(11):522–32. doi: 10.1111/tra.12913 (PMC10946522; doi:10.1111/tra.12913)
Supplement: Supplementary file 1 — Data S1. Supporting information. [file TRA-24-522-s001.docx]

Supplementary Materials for

**Statistical modeling of mRNP transport in dendrites:**

**A Comparative analysis of β-actin and Arc mRNP dynamics**

Hyerim Ahn^1,5^, Xavier Durang^2,5^, Jae Youn Shim^3^, Gaeun Park^3^, Jae-Hyung Jeon^2*^, Hye Yoon Park^1,3,4*^

^1^Department of Electrical and Computer Engineering, University of Minnesota, Minneapolis 55455, USA

^2^Department of Physics, Pohang University of Science and Technology, Pohang 37673, Republic of Korea

^3^Department of Physics and Astronomy, Seoul National University, Seoul, 08826, Republic of Korea

^4^Institute of Applied Physics, Seoul National University, Seoul, 08826, Republic of Korea

^5^These authors contributed equally to this work.

*Corresponding Authors: Jae-Hyung Jeon, [jeonjh@postech.ac.kr](mailto:jeonjh@postech.ac.kr), Hye Yoon Park, [hyp@umn.edu](mailto:hyp@umn.edu)

**This file includes:**

Figure S1 (associated with Figure 3)

Figure S2 (associated with Figure 4)

Figure S3 (associated with Figure 5)

Figure S4 (associated with Figure 5)


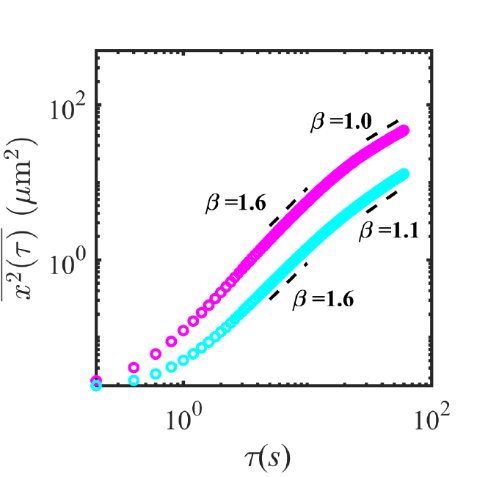


**Figure S1. TA MSD curves of the experimental data for β-actin (cyan) and Arc (magenta) mRNPs**. The two curves have a similar shape, but different scales.


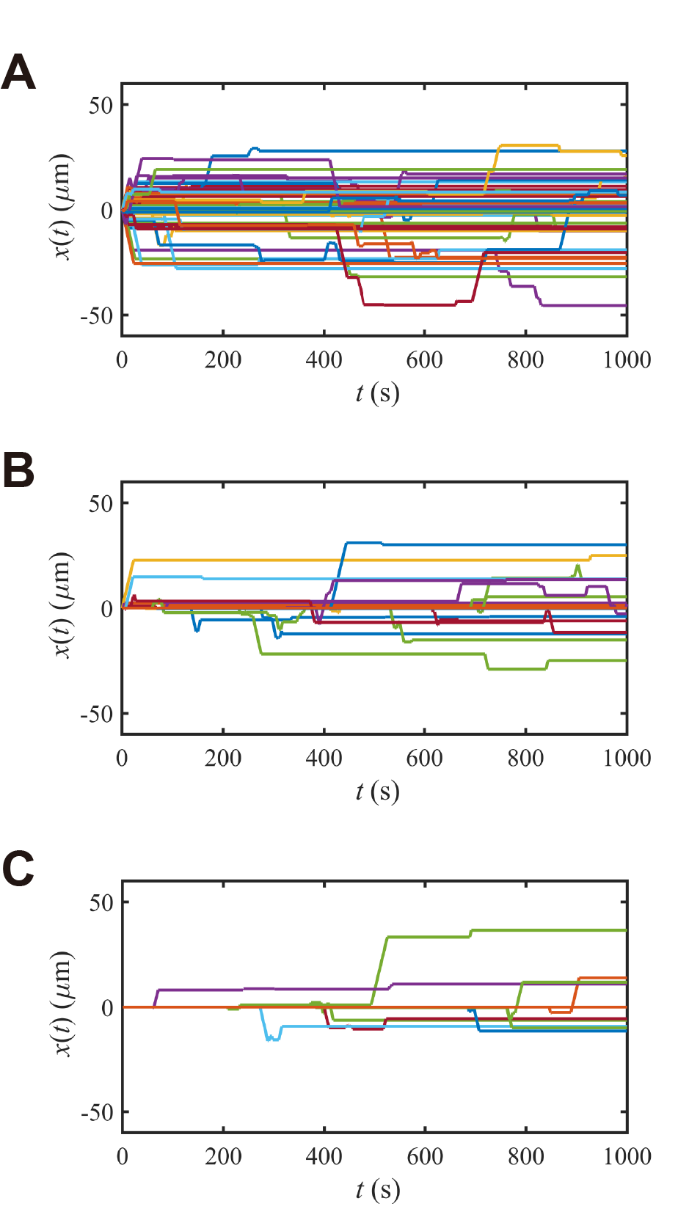


**Figure S2. Simulated trajectories of mRNPs with three different aging times (**$\boldsymbol{t}_{\boldsymbol{a}}$**= 0, 100, 1000 s) for the measurement time *T* = 1000 s.** Each panel shows 50 trajectories randomly selected from 50000 trajectories. As the aging time increases, the number of stationary trajectories increases.


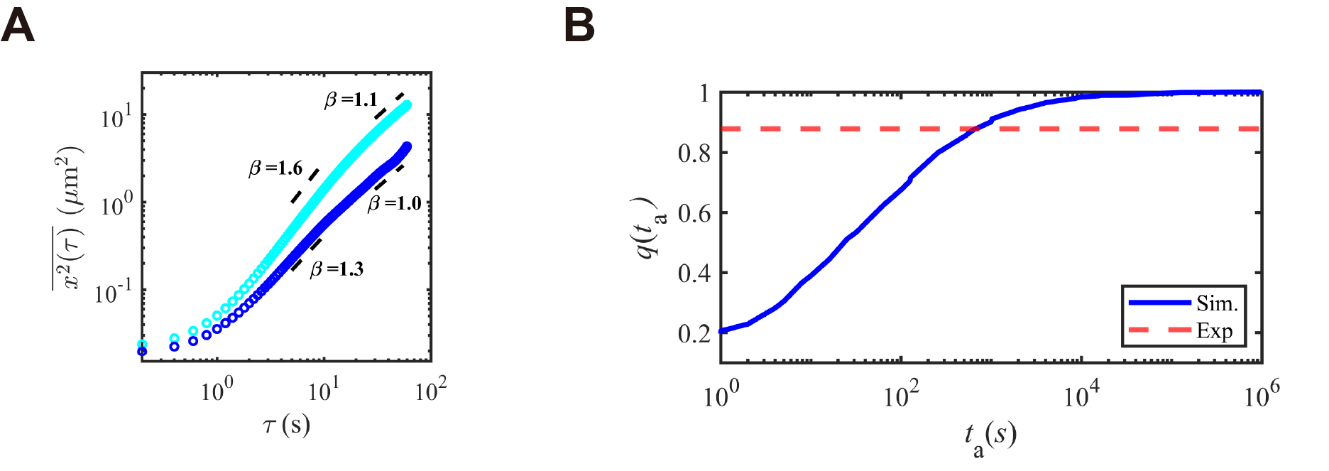


**Figure S3. Aging time of β-actin mRNP in unstimulated neruons. (A)** TA MSD curves of the experimental data for β-actin mRNPs in stimulated (cyan) and unstimulated neurons (blue). **(B)** Fraction of the trajectories showing no run during [$t_{a}$, $t_{a}$+*T*] as a function of $t_{a}$ for β-actin mRNPs in unstimulated neurons. Blue solid line represents the simulation curve and red dashed line indicates the corresponding experimental value (88%).


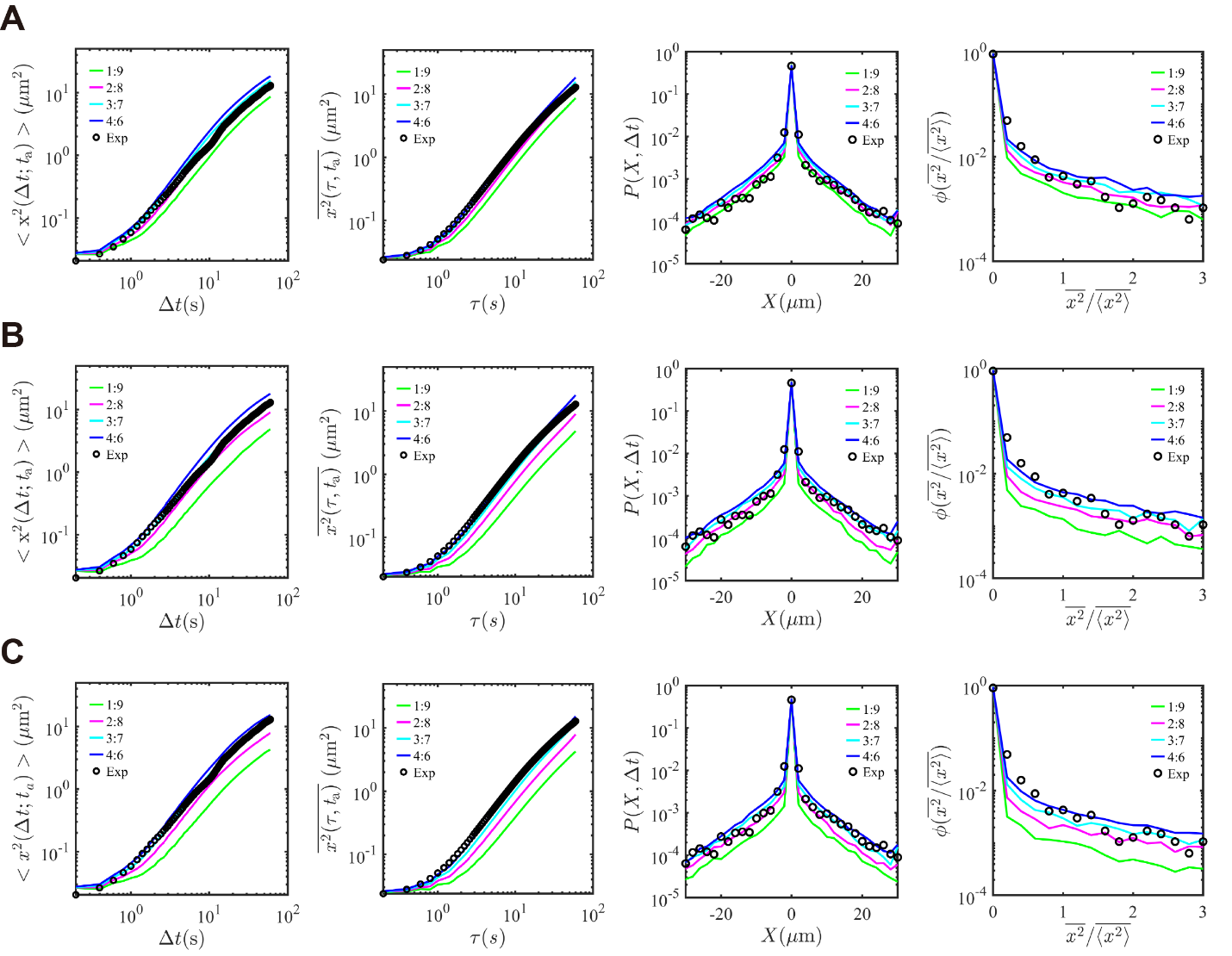


**Figure S4. Simulation results with different aging times of pre-existing β-actin mRNP.** **(A-C)** Comparison of the experimental data for β-actin mRNP (black circles) and the simulation data consisting of two populations with different ratios between newly generated mRNP ($t_{a}$ = 30 s) and pre-existing mRNP ($t_{a}$ = 10 min for (A), 1 hr for (B), and 10 hr for (C)). The first column, EA MSD curves; the second column, TA MSD curves; the third column, the aged probability density functions $P(X, \Delta t=30 s)$; the last column, the scatter distribution function $\phi\left( \bar{x^{2}}/\left\langle\bar{x^{2}} \right\rangle\right)$ of the TA MSD. The ratios between newly formed and pre-existing mRNPs are 1:9, 2:8, 3:7, and 4:6 (green, magenta, cyan and blue curves, respectively).
